# Bi-exponential modelling of $W^{^{\prime}}$ reconstitution kinetics in trained cyclists

**DOI:** 10.1007/s00421-021-04874-3

**Published:** 2021-12-18

**Authors:** Alan Chorley, Richard P. Bott, Simon Marwood, Kevin L. Lamb

**Affiliations:** 1 Department of Sport and Exercise Sciences, University of Chester, Chester, CH1 4BJ UK; 2 School of Health Sciences, Liverpool Hope University, Liverpool, L16 9JD UK

**Keywords:** Critical power, Recovery, $W^{^{\prime}}$, Modelling, Fatigue

## Abstract

**Purpose:**

The aim of this study was to investigate the individual $W^{^{\prime}}$ reconstitution kinetics of trained cyclists following repeated bouts of incremental ramp exercise, and to determine an optimal mathematical model to describe $W^{^{\prime}}$ reconstitution.

**Methods:**

Ten trained cyclists (age 41 ± 10 years; mass 73.4 ± 9.9 kg; $\dot{V}{\text{O}}_{2\max }$ 58.6 ± 7.1 mL kg min^−1^) completed three incremental ramps (20 W min^−1^) to the limit of tolerance with varying recovery durations (15–360 s) on 5–9 occasions. $W^{^{\prime}}$ reconstitution was measured following the first and second recovery periods against which mono-exponential and bi-exponential models were compared with adjusted *R*^2^ and bias-corrected Akaike information criterion (AICc).

**Results:**

A bi-exponential model outperformed the mono-exponential model of $W^{^{\prime}}$ reconstitution (AICc 30.2 versus 72.2), fitting group mean data well (adj*R*^2^ = 0.999) for the first recovery when optimised with parameters of fast component (FC) amplitude = 50.67%; slow component (SC) amplitude = 49.33%; time constant (*τ*)_FC_ = 21.5 s; *τ*_SC_ = 388 s. Following the second recovery, *W*′ reconstitution reduced by 9.1 ± 7.3%, at 180 s and 8.2 ± 9.8% at 240 s resulting in an increase in the modelled *τ*_SC_ to 716 s with *τ*_FC_ unchanged. Individual bi-exponential models also fit well (adj*R*^2^ = 0.978 ± 0.017) with large individual parameter variations (FC amplitude 47.7 ± 17.8%; first recovery: (*τ*)_FC_ = 22.0 ± 11.8 s; (*τ*)_SC_ = 377 ± 100 s; second recovery: (*τ*)_FC_ = 16.3.0 ± 6.6 s; (*τ*)_SC_ = 549 ± 226 s).

**Conclusions:**

W′ reconstitution kinetics were best described by a bi-exponential model consisting of distinct fast and slow phases. The amplitudes of the FC and SC remained unchanged with repeated bouts, with a slowing of W′ reconstitution confined to an increase in the time constant of the slow component.

## Introduction

The critical power model introduced by Monod and Scherrer (1965) describes the hyperbolic relationship between constant power output and tolerable duration within the confines of the ‘severe’ intensity domain (Eq. 1). The model consists of two parameters: critical power (CP), which is the asymptote of the hyperbola, and the curvature constant (W′). Furthermore, the model can also be rearranged mathematically (Morton 1994) to predict the tolerable duration of ramp exercise (Eq. 2) where the S is the ramp rate.

1 $$T_{\lim } = \, W^{^{\prime}} /\left( {P \, - {\text{ CP}}} \right)$$

2 $$T_{\lim } = {\text{CP}}/S + \sqrt {\left( {2 \, \times \, W^{^{\prime}} \, / \, S} \right)} ,$$

where *T*_lim_ is the time to limit of tolerance (s); *W*′ is the work capacity above CP (J); *P* is the power output (W); CP is the critical power (W); *S* is the ramp rate (W s^−1^).

CP represents the highest power output that can be sustained by the provision of adenosine triphosphate from wholly aerobic means (Coats et al. 2003; Poole et al. 1988), and the maximum work rate at which metabolic homeostasis can be maintained. As such, it denotes the physiological boundary between the ‘heavy’ and ‘severe’ intensity domains (Jones et al. 2019). W′ is the finite capacity of work that can be performed above CP (Jones and Vanhatalo 2017), However, the underlying biochemistry that comprises W′ remains only partially understood. Initially thought of as ‘anaerobic work capacity’ and believed to be dependent upon levels of phosphocreatine (PCr), stored glycogen and oxygen within the muscle (Moritani et al. 1981), W′ is now considered to be at least partly dependent upon the accumulation of fatiguing metabolites such as adenosine diphosphate, inorganic phosphates and hydrogen ions (Fukuba et al. 2003; Johnson et al. 2014; Jones et al. 2008). Most recently, exercise-based investigations have associated the magnitude of *W*′ with the development of the oxygen uptake ($\dot{V}{\text{O}}_{2}$) slow component (Burnley and Jones 2018; Murgatroyd et al. 2011), muscle glycogen availability (Clark et al. 2019; Miura et al. 2000), and leg morphology (Byrd et al. 2017). The kinetics of W′ are of particular interest within competitive cycle sport as the outcomes of many races are decided by the efficacy of riders’ intermittent efforts above CP interspersed with short recovery periods below CP (Craig and Norton 2001; Vogt et al. 2007) that allow for the partial reconstitution of W′ (Chidnok et al. 2013a).

Like W′, intramuscular PCr stores deplete when exercising above CP and reconstitute when power output is reduced below CP (Chidnok et al. 2013b). Indeed, there is a significant relationship between the two (Chidnok et al. 2013a), albeit that *W*′ recovers at a slower rate than PCr (Ferguson et al. 2010). Furthermore, both PCr (Chidnok et al. 2013a) and *W*′ reconstitution kinetics (Chorley et al. 2019) are slowed following repeated severe intensity efforts that culminate at the limit of tolerance, suggesting that W′ reconstitution processes are partially dependent upon PCr regeneration. The time-course of *W*′ reconstitution has been described as curvilinear by Ferguson et al. (2010) following observations of its recovery to 37%, 65% and 86% of baseline levels resulting from respective recovery durations of 2, 6 and 15 min. More extensive modelling of *W*′ reconstitution was subsequently carried out by Skiba et al. (2012) to produce a mono-exponential model of *W*′ reconstitution (Eq. 3) derived from a short intermittent exercise protocol (60 s work, 30 s recovery) to the limit of tolerance using untrained cyclists:

3 $$W_{{{\text{bal}}}}^{^{\prime}} = W^{\prime} - \mathop \smallint \limits_{0}^{t} \left( {W_{\exp }^{^{\prime}} } \right) \times\left( {e^{{ - \left( {t - u} \right)/\tau_{{w^{\prime}}} }} } \right),$$

where *W*′_bal_ is the balance of *W*′ at time *t* (J); W′ is the work capacity above CP (J); *W*′_exp_ is the *W*′ expended (J); *t* − *u* is the recovery duration (s); *τ*_*W*′_ is the *W*′ reconstitution time constant (s).

The time constant (*τ*_*W*′_) was found to be inversely correlated to the difference between CP and recovery power output (*D*_CP_) and fitted to the model via non-linear regression to produce Eq. 4 (Skiba et al. 2012).

4 $$\tau_{W\prime } = \, 546 \, \times \, e^{{\left( { - 0.01 \, D_{{{\text{CP}}}} } \right)}} + \, 316,$$

where *τ*_*W*′_ is the *W*′ reconstitution time constant (s); DCP is the difference between CP and recovery power output.

It has, however, been suggested that the model underestimates *W*′ reconstitution in elite cyclists (Bartram et al. 2017) and does not account for the slowing of W′ reconstitution with repeated maximal incremental exercise (Chorley et al. 2019). Furthermore, large individual variations in *τ*_*W*′_ were observed in both the modelling of *τ*_*W*′_ in untrained cyclists (Skiba et al. 2012), and the modified τ_W′_ model for elite cyclists (Bartram et al. 2017). As other research into *W*′ reconstitution kinetics has similarly found high inter-individual variability of W′ reconstitution, it has been argued that *τ*_*W*′_ should be determined on an individual basis (Caen et al. 2019; Chorley et al. 2019; Skiba et al. 2015) rather than the use of Eq. 4. This reliance solely upon *D*_CP_ for the determination of *τ*_*W*′_ has been questioned (Chorley and Lamb 2020) following significant differences being found between predicted *W*′ reconstitution and experimental measurements (Chorley et al. 2019; Lievens et al. 2021) and several markers of aerobic fitness together with age and body composition have been correlated with W′ reconstitution (Chorley et al. 2020). Hence, it is posited that such individual factors might contribute to the accuracy of *W*′ reconstitution modelling.

The time course of *W*′ reconstitution has yet to be completely elucidated, such that it remains unknown whether a mono-exponential or a multi-exponential model best describes *W*′ reconstitution kinetics, and accounts for its slowing due to repeated efforts. Therefore, the main aim of this study was to investigate the individual *W*′ reconstitution kinetics of trained cyclists, specifically over several short duration (< 6 min) time points following repeated maximal incremental exercise, and to determine an optimal non-linear model to describe *W*′ reconstitution. It was hypothesised that W′ reconstitution will be best explained by a multi-exponential mathematical model incorporating variables that account for high inter-individual variations of the *W*′ reconstitution time-course. The secondary aim of the study was to determine if the *W*′ reconstitution model parameters could be adequately determined via fewer (two) exercise testing sessions.

## Methods

### Participants

Following institutional ethical approval, ten adult cyclists (male = 9; female = 1; age 41 ± 10 years; stature 176.6 ± 6.1 cm; body mass 73.4 ± 9.9 kg; $\dot{V}{\text{O}}_{2\max }$ 58.6 ± 7.1 mL kg min^−1^) volunteered to participate in the study and provided written informed consent. Participants were all amateur competitive cyclists (with a training history of 5–14 h week^−1^ for a minimum of 12 months) and familiar with maximal effort testing sessions. Their involvement with the study occurred at the end of their racing seasons.

### Experimental design

**Fig. 1:**
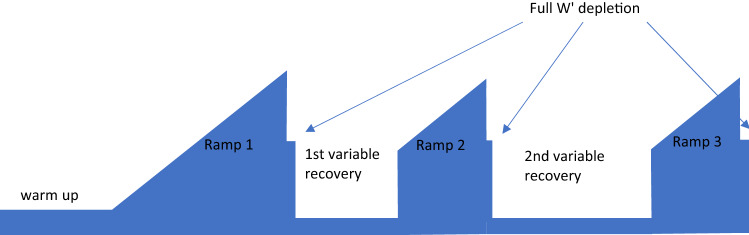
Simplified protocol schematic of the experimental trials, where $W^{^{\prime}}$ is fully depleted during each ramp before an active recovery period allows partial $W^{^{\prime}}$ reconstitution, the extent of which is calculated as the work above critical power in the subsequent depletion

Participants completed between six and ten testing sessions over a maximum period of 3 weeks (6 visits) or 4 weeks (10 visits), with at least 2 days between visits. All sessions were completed in an air-conditioned laboratory (temperature 18.5 ± 1.5 °C) at the same time of day (± 0.75 h). Participants undertook each session having avoided strenuous exercise and alcohol consumption for 24 h, caffeine for 4 h, and were 3 h post-prandial. Visit one incorporated anthropometric and baseline measures of CP and W′, and a familiarisation of the physiological testing procedures employed in the subsequent trials. In the following visits (see Fig. 1) participants completed a repeated ramp cycle test (Chorley et al. 2019) with two varying recovery durations. Recovery durations were paired such that experimental trials comprised the following arrangement for the first and second recovery periods: 30 s and 240 s; 60 s and 180 s; 120 s and 120 s; 180 s and 60 s; 240 s and 60 s. Trial order was randomised and balanced using a Latin square design, ensuring that all durations were undertaken as both a first and second recovery. Three of the participants undertook further sessions (recovery durations: 15 s, 45 s, 5 min, 6 min) to provide an additional individual granularity for use in the modelling of W′ reconstitution. All cycling bouts were performed on an electronically braked ergometer (Lode Excalibur Sport, Lode BV, Groningen, Netherlands), adjusted for each participant and replicated for all visits. Participants were instructed to remain seated during the tests and were equipped with a wireless ANT + chest strap (Garmin International, Kansas, US) for continuous monitoring of heart rate. Pulmonary gas exchange was sampled breath-by-breath to determine O_2_ and CO_2_ concentrations and volumes with an on-line analyser (Quark CPET, Cosmed, Rome, Italy), calibrated prior to each test with gases of known concentrations and volumes.

### Procedures

#### Anthropometric assessments

Stature, body mass, and body composition were recorded via an air displacement plethysmography procedure (BodPod, Life Measurement Instruments, Concord, CA, US; calibrated prior to each visit). Fat mass and fat-free mass were determined from the conversion of estimates of body density via the Siri equation (Siri 1956). Skinfold thicknesses was measured at the right thigh (midway between the inguinal crease and the proximal border of the patella) to 0.1 mm using Harpenden callipers (British Indicators, Luton, UK). Thigh girth was measured at the same position using an anthropometric tape to 0.1 cm. Corresponding muscle girths were calculated from Eq. 5 (Nevill et al. 2003):

5 $${\text{Muscle girth }} = {\text{ leg girth }}{-} \, \left( {\pi \, \times {\text{ skinfold thickness}}} \right).$$

#### Baseline physiological testing

A single test was used to determine CP and to provide a familiarisation for the subsequent experimental protocols. For the accurate determination of CP, the ergometer was configured with a ‘linear factor’ based on estimated CP and preferred cadence derived from participants’ training data (Chorley et al. 2019). Participants cycled for 5 min before transitioning to a 20 W·min^−1^ ramp to the limit of tolerance (with strong verbal encouragement provided) denoted by cadence falling below 60 r min^−1^, at which point power output was automatically stepped down to 30 W above predicted CP to ensure full depletion of *W*′ until cadence fell below 50 r min^−1^, upon which the ergometer immediately switched from hyperbolic mode into linear mode during which they cycled all-out for 2 min. Knowledge of time to completion of this phase was withheld to minimise the likelihood of pacing. For familiarisation a recovery period of 2 min at 50 W preceded a further 20 W·min^−1^ ramp to the limit of tolerance which commenced at CP + 30 W to reduce errors associated with inter-day variability of CP (Chorley et al. 2019). Again, when cadence dropped below 60 r min^−1^ power output was stepped down to CP + 30 W until cadence fell below 50 r min^−1^ and the session was ended.

#### Experimental trials

A repeated ramp test protocol was used for the determination of *W*′ reconstitution. This commenced with 5 min of cycling at 100 W which was below the gas exchange threshold and so within the moderate intensity domain (Coats et al. 2003) before transitioning into a 20 W·min^−1^ ramp to the limit of tolerance, at which point power output was reduced via a step-down to CP + 30 W to ensure full depletion of *W*′. Following this, the first recovery period at a moderate intensity of 50 W was followed by a second ramp commencing at CP + 30 W and again stepping down to CP + 30 W at the limit of tolerance. A second recovery period at 50 W and third ramp and step-down ensued. All ramps increased at a rate of 20 W·min^−1^. The limit of tolerance during ramp phases was denoted by cadence dropping below 60 r min^−1^ and the step-down phase ended when cadence dropped below 50 r min^−1^.

#### Data processing

Errant breaths where removed from gas exchange data where $\dot{V}{\text{O}}_{2}$ differed from the local mean by ≥ 4 SD (Lamarra et al. 1987) before being interpolated and time aligned to power output to produce second-by-second data for the trial using custom spreadsheets in Microsoft Excel (2016). Maximal oxygen uptake ($\dot{V}{\text{O}}_{2\max }$) was deemed to be the maximum mean $\dot{V}{\text{O}}_{2}$ recorded over a 30-s period across all tests (Day et al. 2003). CP was calculated as the mean power output during the final 30 s of the all-out phase of the baseline test (Murgatroyd et al. 2014), and *W*′ as the mean amount of work done above CP during the first ramp and step-down phase over the series of tests. The amount of *W*′ reconstituted during each individual recovery period was calculated as the amount of work completed above CP during the subsequent ramp and step-down phase. %*W*′_rec1_ and %*W*′_rec2_ denote the percentage of *W*′ reconstitution relative to initial W′ arising from the first and second recovery periods, respectively. Heart rate recovery and $\dot{V}{\text{O}}_{2}$ recovery were noted as the difference in absolute heart rates and $\dot{V}{\text{O}}_{2}$ from the end of the *W*′ expenditure to the end of the subsequent recovery period. Excess post-exercise oxygen consumption (EPOC) was the total $\dot{V}{\text{O}}_{2}$ consumed following the end of the ramp-step-down phase for the given recovery duration.

#### Model selection

Mono-exponential and multi-exponential models of W′ reconstitution were generated with OriginPro 2020b (Originlab Corp., Northampton, MA, USA). Multi-exponential models were constrained such that the sum of the amplitude parameters = 100%. Mono-exponential (Eq. 6) and bi-exponential (Eq. 7) models were successfully fitted against the experimental data, and as a tri-exponential model failed to converge, further iterations were not attempted. The two exponential terms of the bi-exponential model are hereafter referred to as the fast component (FC) and slow component (SC). A model comparison was undertaken on two forms of the bi-exponential model against the mean W′ reconstitution experimental data, where the amplitudes of the fast and slow components were either (a) fitted and shared between the first and second recovery periods, or (b) fitted individually for the first and second recovery periods. τ parameters were individually optimized for each recovery period in both cases.

6 $${\text{Mono}} - {\text{exponential}}:\% W_{{{\text{rec}}}}^{^{\prime}} = 100 \times \left( {1 - e^{{ - t/\tau_{{{\text{mono}}}} }} } \right),$$

7 $$\begin{gathered} {\text{Bi}} - {\text{exponential}}: \% W_{{{\text{rec}}}}^{^{\prime}} = {\text{FC}}_{{{\text{amp}}}} \times \left( {1 - e^{{ - t/\tau_{{{\text{FC}}}} }} } \right) + {\text{SC}}_{{{\text{amp}}}} \times \left( {1 - e^{{ - t/\tau_{{{\text{SC}}}} }} } \right) \hfill \\ {\text{and where}} : {\text{SC}}_{{{\text{amp}}}} = (100 - {\text{FC}}_{{{\text{amp}}}} ); \hfill \\ \end{gathered}$$

%*W*′_rec_ is the % of *W*′ reconstituted; *τ* is the *W*′ reconstitution time constant (s) for mono, fast component and slow components, respectively; *t* is the recovery duration(s); *Amp* is the amplitude of fast component and slow component, respectively. The mono-exponential function has two parameters, and the bi-exponential function has five parameters.

Bias-corrected Akaike information criterion (AICc) showed the increased complexity of the model where amplitudes were fitted individually did not improve the fit of the model. The bi-exponential model, where the amplitude was fitted and shared between the first and second recovery periods, was thus chosen to compare in detail with the mono-exponential model seen in previous literature (Skiba et al. 2012, 2015) on mean and individual data. Parameters within the models were fitted using a least squares method via the non-linear curve fitting tool within Origin Pro and assessed using adjusted *R*^2^ and compared using AICc.

### Statistical analysis

Descriptive statistics (mean ± SD) were calculated for all the dependent variables and the normality of their distributions was checked using the Shapiro–Wilk test. Statistical significance was set at *P* < 0.05 throughout. Two-way repeated-measures ANOVA was performed to assess the interactions of recovery duration and recovery phase on *W*′ reconstitution. Sphericity was checked with Mauchly’s test and accounted for where necessary using the Greenhouse–Geisser adjustment. A priori paired sample *t* tests were used to compare the means of *W*′ reconstitution at each time point from the first and second recovery periods, together with effect sizes (ES) calculated as the difference between the means divided by the pooled SD. Pearson’s product–moment correlation coefficients were used to examine the relationships between the parameters of the best-fit model and physiological and anthropometric measurements. Partial correlations (accounting for amplitude bias) were used to assess the relationships between time constant(s) and *W*′ reconstitution at each experimental time point. Linear regression was then performed using data from the time points with the strongest relationships to produce prediction equations for model parameters from a maximum of two test sessions. These equations were assessed against the experimental data using the non-linear curve fitting as previously described. All statistical analyses were performed using SPSS v.26 (IBM Corp., Armonk, NY, US).

## Results

Individual measurements of CP, *W*′, $\dot{V}{\text{O}}_{2\max }$, body mass, and fat mass are shown in Table 1.

**Table 1:** Individual and group (mean ± SD) physiological measurements

Participant	Critical power (W)	*W*′ (kJ)	$\dot{V}{\text{O}}_{2\max }$ (mL kg min^−1^)	Body mass (kg)	Fat mass (%)
1	315	8.5	62.9	63.5	18.3
2	290	10.3	60.7	67.7	14.6
3	338	8.2	71.8	67.1	19.7
4	268	7.7	55.1	66.8	5.1
5	281	11.8	56.3	70.5	18.6
6	296	7.2	50.5	89.5	27.1
7	311	7.3	67.5	66.9	19.0
8	340	10.6	56.7	79.8	17.6
9	337	12.1	55.4	91.0	18.4
10	222	9.9	49.3	71.5	24.9
Mean	300 ± 37	9.4 ± 1.8	58.6 ± 7.1	73.4 ± 9.9	18.3 ± 6.2

### *W*′ reconstitution

**Fig. 2:**
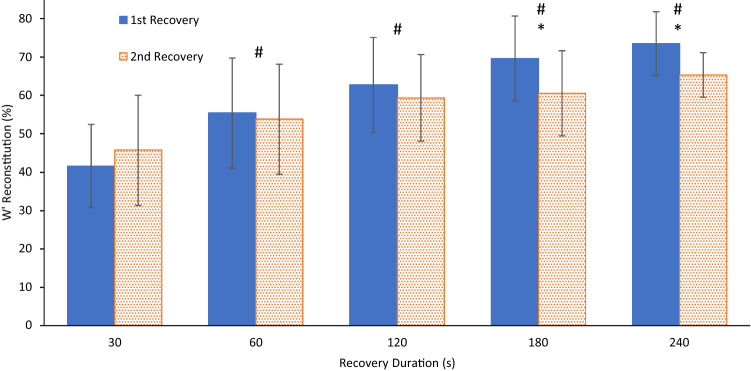
Mean ± SD proportion of $W^{^{\prime}}$ reconstitution during the first and second recovery periods across five recovery durations. *Main effect recovery order (pairwise analysis, *p* < 0.05). ^#^Main effect recovery duration (pairwise analysis, versus previous recovery duration, *p* < 0.01)

The recovery duration range of 30–240 s demonstrated a curvilinear *W*′ reconstitution profile ranging from 41.6 ± 10.8% to 73.5 ± 8.3% (1st recovery) and 45.7 ± 14.3% to 65.3 ± 5.8% (2nd recovery) of initial W′. Significant main effects of recovery duration (*p* < 0.001) and recovery order (*p* = 0.02) on W′ reconstitution were observed, values being higher as recovery duration increased, and lower in the second recovery bout from 180 s onwards (Fig. 2). The interaction effect was also significant (*p* = 0.004), with planned comparisons revealing a non-significant increase in W′ reconstitution between first and second 30-s recovery bouts of 4.2 ± 6.6% (ES = 0.63), but a decreased W′ reconstitution at each of the four longer bouts thereafter (60 s, 1.6 ± 9.5%, ES = 0.16; 120 s, 3.4 ± 6.7%, ES = 0.51; 180 s, 9.1 ± 7.3%, ES = 1.25; 240 s, 8.2 ± 9.8%, ES = 0.84). Mean W′ reconstitution across bouts increased with duration between all the time points (30–60 s, 10.9 ± 10.2%, *p* < 0.001, ES = 1.10; 60–120 s, 6.4 ± 10.3%, *p* = 0.01, ES = 0.59; 120–180 s, 4.1 ± 10.2%, *p* = 0.09, ES = 0.40; 180–240 s, 4.4 ± 8.2%, *p* = 0.03, ES = 0.53).

### Model assessments

Comparison of the bi-exponential model variations where the FC and SC amplitudes were either shared or variable between the first and second recoveries resulted in the preference for the shared amplitude parameter model for the group mean data (shared AICc = 17.0 versus variable AICc = 67.7). Similarly, paired sample *t* tests on the individual fit data also showed a preference for the shared amplitude model shared (AICc = 62.2 ± 17.3 versus variable AICc = 85.5 ± 32.8; *p* = 0006), with no differences in fit (shared adjusted *R*^2^ = 0.978 ± 0.017; variable adjusted *R*^2^ = 0.976 ± 0.020; *p* = 0.06). Therefore, the shared amplitude model was selected as the bi-exponential model to be used in subsequent analysis.

Adjusted *R*^2^ showed the bi-exponential model was a better fit than the mono-exponential model for individual cases (bi-exponential: 0.977 ± 0.017; mono-exponential: 0.740 ± 0.134; *p* < 0.001) when fitted for each participant. However, whilst AICc did not exhibit a difference (*p* = 0.46) for the individual fits between the five-parameter bi-exponential model (62.2: ± 17.3) and the two-parameter mono-exponential model (69.4 ± 13.8), it did so for six out of ten participants including all three participants who completed nine experimental sessions thus generating a greater number of data points for the modelling process. Large inter-individual differences (see Table 2) were evident across all model parameters in both models (bi-exponential: FCamp = 47.7 ± 17.8%; SCamp = 53.3 ± 17.8%; *R*_1_*τ*_FC_ = 22.0 ± 11.8 s; *R*_1_*τ*_SC_ = 377 ± 100 s *R*_2_*τ*_FC_ = 16.6 ± 6.6 s; *R*_2_*τ*_SC_ = 549 ± 226 s mono-exponential: *R*_1_*τ* = 125 ± 53 s; *R*_2_*τ* = 131 ± 69 s).

**Table 2:** Individual best-fit parameters and fit statistics for the mono-exponential and bi-exponential model of W′ reconstitution

Participant	Recovery period	Mono-exponential			Bi-exponential					
		*τ*_mono_ (s)	AICc	Adjusted *R*^2^	FC_Amp_ (%)	SC_Amp_ (%)	*τ*_fc_ (s)	*τ*_sc_ (s)	AICc	Adjusted *R*^2^
1	1	92	59.5	0.697	64.9	35.1	16.3	468	69.0	0.987
	2	50					10.0	552		
2	1	90	57.2	0.800	54.2	45.8	25.8	327	69.1	0.948
	2	116					28.9	686		
3^*^	1	230	84.9	0.834	38.2	61.8	46.3	517	54.6	0.982
	2	163					23.7	349		
4	1	75	64.3	0.643	60.4	39.6	8.2	443	54.5	0.989
	2	110					18.9	861		
5	1	122	84.1	0.458	45.5	54.5	17.2	390	33.4	0.994
	2	211					14.3	980		
6	1	76	50.3	0.877	73.0	27.0	31.5	488	98.8	0.997
	2	37					16.8	445		
7	1	90	62.2	0.736	56.2	43.8	30.8	321	70.1	0.950
	2	64					16.4	457		
8^a^	1	181	74.1	0.934	13.5	86.5	10.1	228	66.6	0.972
	2	230					17.1	294		
9^a^	1	175	92.4	0.701	29.6	70.4	12.3	323	47.2	0.988
	2	199					8.1	372		
10	1	114	64.9	0.719	41.9	58.1	21.2	260	58.5	0.972
	2	128					8.5	493		
Mean	1	125 ± 53	69.4 ± 13.8	0.733 ± 0.14	47.7 ± 17.8	52.3 ± 17.8	22.0 ± 11.8	377 ± 100	62.2 ± 17.3	0.978 ± 0.017
	2	131 ± 69					16.3 ± 6.6	549 ± 226		
*										

^a^Participants undertook additional experimental sessions (totalling 9) covering 15–360 s of recovery

**Fig. 3:**
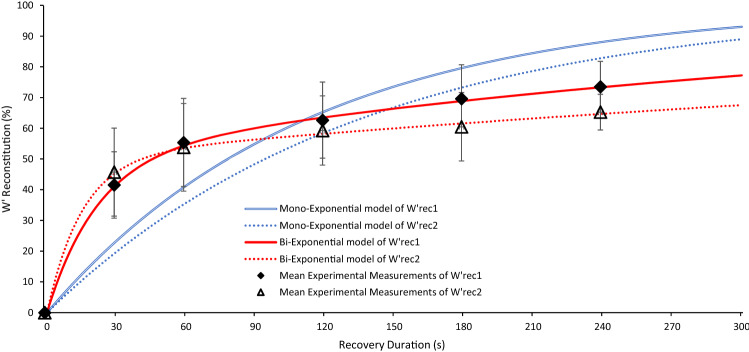
Optimised fit of mono-exponential and bi-exponential models on the group mean *W*′ reconstitution following the first and second recovery periods. Error bars represent ± SD of the mean

When fitted against the group mean data (see Fig. 3), the shared amplitude bi-exponential model was preferred to the mono-exponential model (shared AICc = 17.0 versus mono AICc = 72.2), with best-fit mono-exponential model parameters of *τ*_mono_ = 112.5 s and 135.7 s for the first and second recovery periods, respectively. The mono-exponential model also demonstrated an inferior goodness of fit (adjusted *R*^2^ = 0.614) when compared to the bi-exponential model (adjusted *R*^2^ = 0.999), when fitted as per Eqs. 8 and 9 (below).

8 $$\% W^{\prime}_{rec1} = 50.67 \times\left( {1 - e^{ - t/21.5} } \right) + 49.33 x \left( {1 - e^{ - t/388} } \right),$$

9 $$\% W^{\prime}_{rec2} = 50.67 \times \left( {1 - e^{ - t/15.1} } \right) + 49.33 x \left( {1 - e^{ - t/716} } \right).$$

**Fig. 4:**
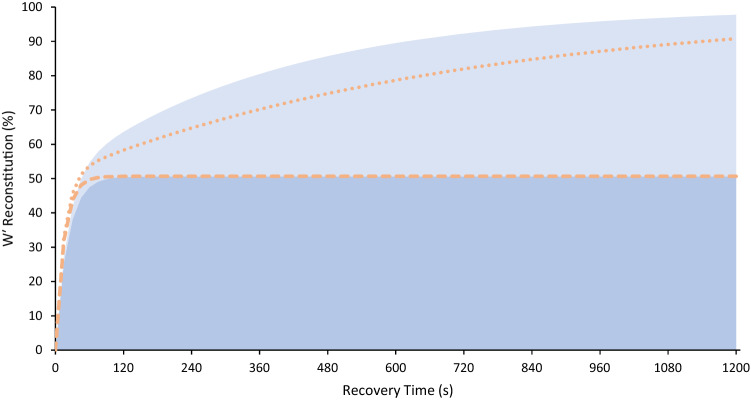
The modelled cumulative contributions of fast (darker) and slow (lighter) components *W*′ reconstitution modelled on the group mean data. Shaded area shows reconstitution following the first recovery, dashed lines following the second recovery

The bi-exponential model of the group mean data demonstrates that the fast component of *W*′ reconstitution is over 90% complete by 50 s, whilst the slow component takes 892 s to attain the 90% level after the first recovery, and 1650 s following the second recovery (see Fig. 4).

### Bi-exponential model parameter relationships

The proportional split of *W*′ reconstitution between the fast component amplitude (FCamp) and slow component amplitudes (SCamp) was significantly correlated to the absolute magnitude of *W*′ such that the greater the absolute *W*′, the greater was the proportion of W′ reconstitution attributed to the slow component; *W*′ was positively correlated to SCamp (*r* = 0.66; *p* = 0.04) and inversely correlated to the FCamp (*r* = – 0.66; *p* = 0.04). FCamp was also strongly related to the fraction of *W*′ reconstituted after 30 s of both the first (*r* = 0.83; *p* < 0.01) and second recovery periods (*r* = 0.87 *p* < 0.01). Heart rate recovery during the first 30 s of the second recovery period was positively related to FCamp *(r* = 0.71; *p* = 0.02), but non-significantly during the first recovery (*r* = 0.37; *p* = 0.29). $\dot{V}{\text{O}}_{2}$ recovery during the first 30 s was similarly related to FCamp (first recovery:* r* = 0.33; *p* = 0.35; second recovery: *r* = 0.63; *p* = 0.05).

**Fig. 5:**
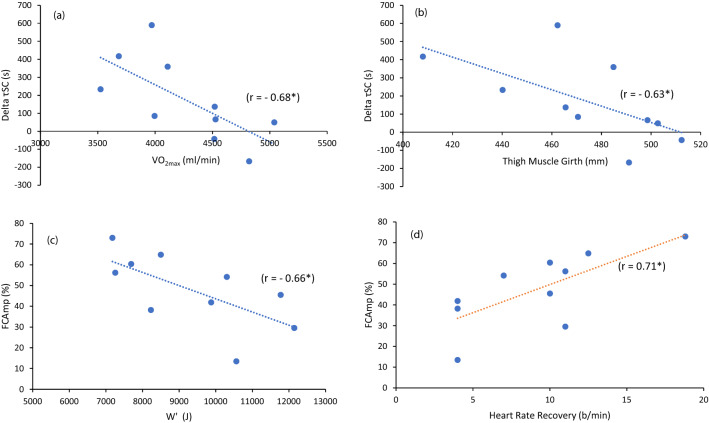
Relationships between physiological measurements and model parameters. **a** $\Delta \tau_{{{\text{SC}}}}$ and absolute $\dot{V}{\text{O}}_{2\max }$. **b** $\Delta \tau_{{{\text{SC}}}}$ and thigh muscle girth. **c** FCAmp and absolute $W^{^{\prime}}$. **d** FCAmp and the difference in heart rate during the second 30 s period. *p < 0.05

There were no differences between *τ*_FC_ derived from the first (*R*_1_*τ*_FC_) and second (*R*_2_*τ*_FC_) recovery periods (22.0 ± 11.8 s versus 16.3 ± 6.6 s; *p* = 0.18), however τ_SC_ increased from the first recovery (*R*_1_*τ*_SC_) to the second recovery (*R*_2_*τ*_SC_) (377 ± 100 s versus 549 ± 226 s; *p* = 0.04). This difference between *R*_1_*τ*_SC_ and *R*_2_*τ*_SC_ ($\Delta \tau_{{{\text{SC}}}}$) was negatively correlated with CP (*r* = – 0.59; *p* = 0.07), $\dot{V}{\text{O}}_{2\max }$ (*r* = – 0.68; *p* = 0.03) and thigh muscle girth (*r* = – 0.63; *p* = 0.05) such that the greater the physiological variable, the smaller the change in *τ*_SC._ Similarly, greater EPOC throughout the recovery period from 30 to 240 s was associated with a $\Delta \tau_{{{\text{SC}}}}$ (*r* > − 0.61; *p* < 0.06). Notable relationships are shown in Fig. 5. No other physiological, body composition or anthropometric correlations of note were detected between for either τ_FC_ or τ_SC._

The magnitude of *W*′ reconstitution at 30 s showed the strongest relationship with *τ*_FC_, being inversely correlated with the fraction of W′ reconstitution of both recovery periods when controlling for FCamp (*R*_1_*τ*_FC_*: r* = – 0.80; *p* = 0.01; *R*_2_*τ*_FC_*: r* = – 0.76; *p* = 0.02) making the fractional reconstitution of *W*′ at 30 s the best predictor of *τ*_FC_. Conversely, *τ*_SC_ was moderately inversely correlated with the fraction of *W*′ reconstitution at the 240-s time points of both recovery periods when controlling for FCamp (*R*_1_*τ*_SC_*: r* = – 0.53; *p* = 0.15; *R*_2_*τ*_SC_*: r* = – 0.62; *p* = 0.07). The linear prediction equations for the parameters of the bi-exponential model from the subset of data available from these two time points (30 and 240 s) were:

10 $$\begin{gathered} {\text{FC}}_{{{\text{amp}}}} = - 0.004 \times W^{\prime} + 1.104 \times R_{1} \_30 + 35.812, \hfill \\ {\text{Recovery}}\;1:{ }\tau_{{{\text{FC}}}} = 0.917 \times {\text{FC}}_{{{\text{amp}}}} - 1.517 \times R_{1} \_30 + 41.239, \hfill \\ {\text{Recovery}}\;1:{ }\tau_{{{\text{SC}}}} = 4.113 \times {\text{FC}}_{{{\text{amp}}}} - 5.17 \times R_{1} \_240 + 559.944, \hfill \\ {\text{Recovery}}\;2:{ }\tau_{{{\text{FC}}}} = 0.548 \times {\text{FC}}_{{{\text{amp}}}} { } - .717 \times R_{2} \_30 + 22.903, \hfill \\ {\text{Recovery}}\;2:{ }\tau_{{{\text{SC}}}} = 10.048 \times {\text{FC}}_{{{\text{amp}}}} - 26.572 \times R_{2} \_240 + 1803.097, \hfill \\ \end{gathered}$$

where *W*′ is the baseline measurement of *W*′; *R*_1__30, *R*_1__240 and *R*_2__240 are the % of *W*′ reconstitution measured after 30 s and 240 s of the first and second recovery periods, respectively.

When retrofit into the bi-exponential model (Eq. 7), using individual measured values for W′ and the fractional reconstitution of *W*′ at 30 s and 240 s, the prediction equations (Eq. 10) proved a successful fit (based on three parameters for recovery 1 and four parameters for recovery 2) against each participant’s W′ reconstitution time course (Recovery 1: adjusted *R*^2^ = 0.958 ± 0.030; Recovery 2: adjusted *R*^2^ = 0.934 ± 0.055).

## Discussion

This study has demonstrated the time-course of W′ reconstitution tracked a curvilinear path for all participants following both the first and second recovery periods, extending to approximately 75% of *W*′ reconstitution within the first 4 min of recovery. Our data mirrors previous findings (Ferguson et al. 2010); however, the additional data over the short (< 2 min) recovery times from the present study revealed a bi-phasic pattern of *W*′ reconstitution kinetics comprising a distinct initial fast phase of W′ reconstitution before noticeably slowing from 60 s onwards. The new bi-exponential model proved to be an excellent fit and superior to existing mono-exponential models of W′ reconstitution. Furthermore, this study demonstrated the novel finding that the fatiguing effect of repeated bouts (Chorley et al. 2019) is confined to the slower phase of W′ reconstitution, evident beyond 180 s (see Fig. 2). That the extent of W′ reconstitution in the current study was notably greater than previously shown by Ferguson et al. (2010) after 120 s of recovery (~ 63% versus ~ 37%) is likely explained by the different training status and the resultant critical power (~ 300 W versus ~ 213 W) of the participants (Chorley et al. 2020; Skiba et al. 2012); however, the effect of the differing ramp and constant load exercise during the W′ depletion phase remains to be determined. Despite the homogeneity of the participants in the present study in terms of CP (CV: 12.4%), *W*′ (CV: 19.6%) and $\dot{V}{\text{O}}_{2\max }$ (CV: 11.5%), the reconstitution of *W*′ (CV: 28.8% at 30 s) varied markedly between individuals, particularly over the shorter durations, with *W*′ reconstitution ranging between 24 and 60% (absolute values: 2.5–4.8 kJ) after the first 30 s. Large differences in *W*′ reconstitution rates have previously been reported via the τ of mono-exponential models (Caen et al. 2019; Skiba et al. 2014b).

We hypothesised that a multi-exponential model would best represent the curvilinear reconstitution of W′ following exhaustive exercise, and indeed when fitted against the measurements of W′ reconstitution the mono-exponential function proved a poor fit, even when individually fitted for each participant, whilst the bi-exponential model proved to fit well when individually parameterised (yielding an adjusted *R*^2^ > 0.94 in all cases). That the bi-exponential model was not preferred in all individual cases based on AICc analysis was likely due to the relatively high number of model parameters of the bi-exponential model compared and relatively low number of W′ reconstitution data points. Indeed, the bi-exponential model was preferred for all those participants completing the additional four test sessions. The *W*′_bal_ models (Skiba et al. 2012, 2015) and modifications (Bartram et al. 2017) previously explored are based on mono-exponential reconstitution of W′, originally devised following an intermittent 60-s work, 30-s recovery protocol to exhaustion, with no intermediate measurements of *W*′ possible. The mono-exponential *W*′_bal_ model has been validated using similar intermittent protocols in hypoxia (Shearman et al. 2016; Townsend et al. 2017), and by retrofitting to the point of exhaustion during training and race data (Skiba et al. 2014a), where the mono-exponential model proved a successful fit against the measurements of W′ reconstitution over the short intermittent recoveries. Validations of the mono-exponential *W*′_bal_ model via different protocols have, however, found significant differences against longer recovery durations (Chorley et al. 2019) and partial prior depletion of *W*′ (Lievens et al. 2021; Sreedhara et al. 2020), albeit without τ being individually fitted. Where *τ* has been individualised, it has only been done so against W′ reconstitution at specific measured time points (Caen et al. 2019; Chorley et al. 2020) rather than against a time-course of *W*′ reconstitution.

Whilst the mono-exponential model can be resolved such that *τ* is adjusted to fit any single time point, it cannot follow the reconstitution of W′ over time as well as the bi-exponential model does since the latter accommodates the compartmental fast and slow phases observed in the *W*′ reconstitution time course. The fast and slow components for both the group fit and the mean of the individual fits are of similar magnitudes (each being ~ 50% of the overall recovery magnitude); however, as with their respective τ there is high variability between individuals. The underlying determinants of W′ were originally thought to comprise phosphate and intramuscular stores of glycogen and oxygen (Moritani et al. 1981), yet later findings have suggested an accumulation of fatiguing metabolites and muscle metabolic perturbations (Jones et al. 2008; Vanhatalo et al. 2016). It would seem plausible that the complex mechanisms that underpin W′ reconstitution are at least partially dependent upon both the replenishment of energy stores and the removal of muscle metabolites. Indeed, the exponential recovery of PCr has been previously evaluated as *τ* = 29.4 s in the vastus lateralis (van den Broek et al. 2007) and *τ* = 25 s in the calf (Haseler et al. 1999) both comparing closely to *τ*_FC_ in the present study, whilst *τ*_SC_ is comparable to blood lactate clearance following a repeated sprint protocol (Menzies et al. 2010). Attributing the fast and slow components of the bi-exponential model to these two factors may be oversimplistic, given that PCr recovery alone may follow a more complex bi-phasic time course (Harris et al. 1976; Iotti et al. 2004), and that blood lactate is at best a delayed proxy for muscle metabolism (Rusko et al. 1986), hence the need for a greater understanding of the interactions that comprise *W*′. As PCr recovery is an oxygen dependent process (Haseler et al. 1999), it is likely that $\dot{V}{\text{O}}_{2}$ kinetics will influence the restoration rate of PCr during recovery and consequently the reconstitution of W′. Prior exercise has been shown to alter $\dot{V}{\text{O}}_{2}$ responses for up to 45 min (Burnley et al. 2006) and repeated sprint performance which is almost certainly dependent upon W′ reconstitution during recovery, is better maintained by those with faster $\dot{V}{\text{O}}_{2}$ kinetics during the recovery phase (Dupont et al. 2010). Whilst detailed $\dot{V}{\text{O}}_{2}$ kinetics were beyond the scope of the current study, the relationship between $\dot{V}{\text{O}}_{2}$ recovery and FCamp suggests further investigation is warranted.

The bi-exponential model presented demonstrates that fatigue due to repeated efforts is confined to the slowing of *W*′ reconstitution kinetics represented by an increase in *τ*. The relative amplitudes of the fast and slow components remain unchanged between the recovery periods following the modelling optimisation process, suggesting that small variations in amplitude do not warrant a more complex model. Given that *W*′ reconstitution has been shown to slow following repeated efforts (Chorley et al. 2019, 2020), that *τ*_FC_ in the present study did not increase was somewhat unexpected. Colloquial cycling terminology refers to cyclists “burning matches” when they perform high-intensity surges and having a limited number of “matches” available; it is feasible that *τ*_FC_ may increase after a greater number of repetitions than undertaken in the present study. Contrastingly, *τ*_SC_ did increase significantly during the recovery period following a single repeated bout. Neither body composition nor physiological characteristics in this homogenous group were found to be related to *τ*_SC_ itself. However, the difference in *τ*_SC_ between the first and the second recoveries ($\Delta \tau_{{{\text{SC}}}}$), which describes the extent of the slowing of *W*′ reconstitution with repeated bouts of exercise, was related to the measures of aerobic fitness (CP and $\dot{V}{\text{O}}_{2\max }$), heart rate recovery and EPOC, as found previously (Chorley et al. 2020). Additionally, the delta τ_SC_ was related to thigh muscle girth, which has previously been shown to correlate with *W*′ (Kordi et al. 2018; Miura et al. 2002) independently of muscle fibre type distribution (Vanhatalo et al. 2016). Interestingly, one participant had a greater *W*′ reconstitution following the second recovery across all time points (15–360 s) which stood out from the correlates of aerobic fitness. Whilst the individual demonstrated high $\dot{V}{\text{O}}_{2\max }$ and CP it was notable that he was alone in having previously competed as an elite road cyclist, indicating that fatigue resistance may have a hitherto unexplained component that influences race performance and selection.

The present study demonstrated a bi-exponential, rather than a mono-exponential, model provides a superior fit to W′ reconstitution kinetics during active recovery at a nominal 50 W. Exponential models have been used to describe physiological processes such as PCr recovery (Iotti et al. 2004; van den Broek et al. 2007) and the goodness of fit of the bi-exponential model supports its selection in the present study. Other mathematical models could also be generated to describe W′ reconstitution kinetics; however, it is likely a larger number of model parameters would be required to do so. A secondary finding was that when the bi-exponential model parameters were calculated using measured W′ and its fractional reconstitution from only the 30-s and 240-s time points, this provided an excellent fit against the W′ reconstitution kinetics for everyone that was no different to that of using multiple recovery time points. That the prediction model produces comparable results from just the 30-s and 240-s time points allows the test burden to be reduced considerably (to a baseline and two experimental tests), although it should be noted that the prediction equations have yet to be tested against a different data set. As the effect of changing recovery power output is known to affect W′ reconstitution below CP (Caen et al. 2019; Skiba et al. 2012), future studies should seek to establish a three-dimensional model that explains W′ reconstitution kinetics at varying recovery power outputs as would be encountered under race conditions, enabling its application to competitive cycle sport.

## Conclusions

Understanding the reconstitution kinetics of *W*′ of individual athletes can describe a performance characteristic which can be used to influence race outcomes tactically by manipulating severe intensity attacks and recovery durations. The present data has shown that the reconstitution kinetics of *W*′ among trained cyclists were best described by a new bi-exponential model based on a fast component and a slow component, the parameters of which varied markedly for individual cyclists despite similar training status. A further novel finding was that the slow component alone exhibited impaired *W*′ reconstitution kinetics following a repeated bout of exercise, the magnitude of which was related to measures of aerobic fitness. The awareness of such individual characteristics can be used to inform training programmes and race tactics. Additionally, for assessment and monitoring purposes, using only 30-s and 240-s recoveries were found to be effective in determining *W*′ reconstitution kinetics when compared to modelling using a wider range of recovery durations.

## Data Availability

Data is available upon request from the corresponding author.

## Abbreviations

AICc: Bias-corrected Akaike information criterion
ANOVA: Analysis of variance
CP: Critical power
CV: Coefficient of variation
*D*_CP_: Difference between CP and recovery power output
ES: Effect Size
FC: Fast component (first phase of bi-exponential model)
EPOC: Excess post-exercise oxygen consumption
PCr: Phosphocreatine
$R_{1}$: First recovery period
$R_{2}$: Second recovery period
SC: Slow component (second phase of bi-exponential model)
$$\dot{V}{\text{O}}_{2}$$: Oxygen uptake
$\dot{V}{\text{O}}_{2\max }$: Maximum oxygen uptake
$W^{^{\prime}}$: The finite capacity of work above critical power
$W_{{{\text{rec}}1}}^{^{\prime}}$: Amount of *W*′ reconstituted during the first recovery period
$W_{{{\text{rec2}}}}^{^{\prime}}$: Amount of *W*′ reconstituted during the second recovery period
$(\Delta \tau_{{{\text{SC}}}} )$: Difference in slow component time constants between the first and second recovery periods
$\tau$: Tau, the time constant of W*′* reconstitution
